# Supplementary material for: Prevalence of Alzheimer’s disease pathology in the community
Source: Nature. 2025 Dec 17;650(8100):182–6. doi: 10.1038/s41586-025-09841-y (PMC12872468; doi:10.1038/s41586-025-09841-y)
Supplement: Supplementary file 1 — Supplementary Methods and references [file 41586_2025_9841_MOESM1_ESM.docx]

***Assessment of cognition, physical performance, anxiety, depression, neuropsychiatric symptoms and activities of daily living***

Trained health personnel assessed the participants’ cognitive, neuropsychiatric, and functional status using standardized clinical assessments at a field station, at homes, or in nursing homes. Cognition was assessed with the Norwegian version of the Montreal Cognitive Assessment (MoCA), a screening tool suitable to detect MCI and mild dementia.^1,2^ The Norwegian version of the Word List Memory Task (WLMT) from the Consortium to Establish a Registry of Alzheimer’s disease (CERAD), testing immediate and delayed memory, was additionally conducted in those participants scoring ≥ 22 on the MoCA, except for those participants not remembering any of the 5 words on MoCA subtask 7.^3^ Participants were also asked to fill out the Meta-Memory Questionnaire (MMQ), a nine-item self-report questionnaire designed to assess subjective memory performance.^4,5^ A structured interview with a family or professional (in nursing homes) was conducted in participants suspected of having significant cognitive impairment, either because of scoring below age-adjusted cut-offs or because of self-reported cognitive decline. For participants living in nursing homes, the Severe Impairment Battery-8 (SIB-8) was used instead of MoCA when the structured interview with the professional caregiver indicated moderate to severe dementia. The SIB-8 is validated for assessing cognition in individuals with moderate to severe dementia.^6^ The overall level of cognitive and functional impairment was assessed with the Clinical Dementia Rating Scale (CDR).^7^

Physical performance was screened with the Short Physical Performance Battery (SPPB).^8^ The Hospital Anxiety and Depression Scale (HADS) was used to screen for anxiety and depression.^9^ Other tools used were the Instrumental Activities of Daily Living Scale (I-ADL),^10^ the Physical Self-Maintenance Scale (PSMS),^10^ and the Neuropsychiatric Inventory Questionnaire (NPI-Q).^11^

***Cognitive diagnosis***

For each participant, two clinical and research experts from a pool of nine geriatricians, neurologists, and old-age psychiatrists made independent diagnoses according to The Diagnostic and Statistical Manual of Mental Disorders, Fifth Edition (DSM-5) criteria. If no consensus was reached between the two experts, a third was consulted. Participants were categorized into cognitively unimpaired, mild cognitive impairment (MCI) (minor neurocognitive disorder in DSM-5) or dementia (major neurocognitive disorder in DSM-5).^12^ Those with MCI were further classified as having amnestic MCI (aMCI), i.e. when the predominant cognitive symptom was memory impairment, or non-amnestic MCI (naMCI), i.e. when other cognitive symptoms than memory impairment were dominant. Those with dementia were classified as having mild, moderate or severe dementia. Furthermore, people with a diagnosis of dementia were categorized as having Alzheimer’s disease, vascular dementia, Lewy body dementia (including dementia with Lewy bodies and Parkinson’s disease dementia), frontotemporal dementia, mixed dementia, other specified dementia, or unspecified dementia. Mixed dementia was defined as dementia due to multiple etiologies in line with the DSM-5 criteria for Major Neurocognitive Disorder due to Multiple Etiologies. The detailed diagnostic procedure has been published previously.^13,14^

Of the 8,949 HUNT4 70+ participants with available blood samples, 153 could not be classified as cognitively unimpaired, MCI, or dementia. This was due to missing information on cognitive status – either refusal to undergo cognitive testing or lack of available caregiver information.

**References**

1. Nasreddine, Z.S.*, et al.* The Montreal Cognitive Assessment, MoCA: a brief screening tool for mild cognitive impairment. *J. Am. Geriatr. Soc.* **53**, 695–699 (2005).

2. Islam, N.*, et al.* Accuracy of the Montreal Cognitive Assessment tool for detecting mild cognitive impairment: A systematic review and meta-analysis. *Alzheimers. Dement.* **19**, 3235–3243 (2023).

3. Morris, J.C.*, et al.* The Consortium to Establish a Registry for Alzheimer's Disease (CERAD). Part I. Clinical and neuropsychological assessment of Alzheimer's disease. *Neurology* **39**, 1159–1165 (1989).

4. Almkvist, O., Bosnes, O., Bosnes, I. & Stordal, E. Selective impact of disease on short-term and long-term components of self-reported memory: a population-based HUNT study. *BMJ Open* **7**, e013586 (2017).

5. Bosnes, O., Almkvist, O., Bosnes, I. & Stordal, E. Subjective working memory predicts objective memory in cognitively normal aging: a HUNT study. *BMC Psychol.* **8**, 77 (2020).

6. Schmitt, F.A., Saxton, J., Ferris, S.H., Mackell, J. & Sun, Y. Evaluation of an 8-item Severe Impairment Battery (SIB-8) vs. the full SIB in moderate to severe Alzheimer's disease patients participating in a donepezil study. *Int. J. Clin. Pract.* **67**, 1050–1056 (2013).

7. Hughes, C.P., Berg, L., Danziger, W.L., Coben, L.A. & Martin, R.L. A new clinical scale for the staging of dementia. *Br. J. Psychiatry* **140**, 566–572 (1982).

8. Olsen, C.F. & Bergland, A. Reliability of the Norwegian version of the short physical performance battery in older people with and without dementia. *BMC Geriatr.* **17**, 124 (2017).

9. Zigmond, A.S. & Snaith, R.P. The hospital anxiety and depression scale. *Acta Psychiatr. Scand.* **67**, 361–370 (1983).

10. Lawton, M.P. & Brody, E.M. Assessment of older people: self-maintaining and instrumental activities of daily living. *Gerontologist* **9**, 179–186 (1969).

11. Kaufer, D.I.*, et al.* Validation of the NPI-Q, a brief clinical form of the Neuropsychiatric Inventory. *J. Neuropsychiatry Clin. Neurosci.* **12**, 233–239 (2000).

12. *American Psychiatric Association: Diagnostic and Statistical Manual of Mental Disorders, Fifth Edition. Arlington, VA, American Psychiatric Association, 2013*.

13. GjØra, L.*, et al.* Current and Future Prevalence Estimates of Mild Cognitive Impairment, Dementia, and Its Subtypes in a Population-Based Sample of People 70 Years and Older in Norway: The HUNT Study. *J. Alzheimers. Dis.* **79**, 1213–1226 (2021).

14. Molvik, I.*, et al.* Incidence of dementia among individuals 70 years and older in Norway: A HUNT study. *J. Alzheimers. Dis.*, 13872877251371242 (2025).
