# Supplementary material for: Prevalence of Alzheimer’s disease pathology in the community
Source: Nature. 2025 Dec 17;650(8100):182–6. doi: 10.1038/s41586-025-09841-y (PMC12872468; doi:10.1038/s41586-025-09841-y)
Supplement: Supplementary file 2 — Reporting Summary [file 41586_2025_9841_MOESM2_ESM.pdf]

nature portfolio

Corresponding author(s): Anita Lenora Sunde

Last updated by author(s): October 17, 2025

Reporting Summary

Nature Portfolio wishes to improve the reproducibility of the work that we publish. This form provides structure for consistency and transparency in reporting. For further information on Nature Portfolio policies, see our Editorial Policies and the Editorial Policy Checklist.

Statistics

For all statistical analyses, confirm that the following items are present in the figure legend, table legend, main text, or Methods section.

n/a	Confirmed	
☐	☒	The exact sample size (n) for each experimental group/condition, given as a discrete number and unit of measurement
☐	☒	A statement on whether measurements were taken from distinct samples or whether the same sample was measured repeatedly
☐	☒	The statistical test(s) used AND whether they are one- or two-sided<br>*Only common tests should be described solely by name; describe more complex techniques in the Methods section.*
☐	☒	A description of all covariates tested
☐	☒	A description of any assumptions or corrections, such as tests of normality and adjustment for multiple comparisons
☐	☒	A full description of the statistical parameters including central tendency (e.g. means) or other basic estimates (e.g. regression coefficient) AND variation (e.g. standard deviation) or associated estimates of uncertainty (e.g. confidence intervals)
☐	☒	For null hypothesis testing, the test statistic (e.g. F, t, r) with confidence intervals, effect sizes, degrees of freedom and P value noted<br>*Give P values as exact values whenever suitable.*
☒	☐	For Bayesian analysis, information on the choice of priors and Markov chain Monte Carlo settings
☒	☐	For hierarchical and complex designs, identification of the appropriate level for tests and full reporting of outcomes
☒	☐	Estimates of effect sizes (e.g. Cohen's d, Pearson's r), indicating how they were calculated

Our web collection on statistics for biologists contains articles on many of the points above.

Software and code

Policy information about availability of computer code

Data collection	No software was used for data collection as part of this specific study.
Data analysis	R version 4.5.0; R Core Team, packages haven, survey, tidyverse, ggplot2, ggpubr, ggbeeswarm, patchwork, segmented. All codes for data processing and analysis are publicly available at https://github.com/dalejo643/Nature-Aarsland2025.git

For manuscripts utilizing custom algorithms or software that are central to the research but not yet described in published literature, software must be made available to editors and reviewers. We strongly encourage code deposition in a community repository (e.g. GitHub). See the Nature Portfolio guidelines for submitting code & software for further information.

Data

Policy information about availability of data

All manuscripts must include a data availability statement. This statement should provide the following information, where applicable:

- Accession codes, unique identifiers, or web links for publicly available datasets
- A description of any restrictions on data availability
- For clinical datasets or third party data, please ensure that the statement adheres to our policy

To protect participants' privacy, HUNT Research Centre aims to limit storage of data outside HUNT databank and cannot deposit data in open repositories. HUNT databank has precise information on all data exported to different projects and can reproduce them on request. There are no restrictions regarding data export given approval of applications to HUNT Research Centre. Researchers can apply for data at https://www.ntnu.edu/hunt/research.

Research involving human participants, their data, or biological material

Policy information about studies with human participants or human data. See also policy information about sex, gender (identity/presentation), and sexual orientation and race, ethnicity and racism.

Reporting on sex and gender	Sex was defined as either female or male according to the status registered in the Norwegian National Registry at the time of invitation. No information on gender was collected.
Reporting on race, ethnicity, or other socially relevant groupings	The HUNT study does not collect data on ethnicity, but the population in this region in 2017 included less than 5% immigrants or Norwegian-born to immigrant parents from Africa, Asia, Middle- or South America, and thus the findings are relevant for a mainly Caucasian Norwegian population. Education was self-reported.
Population characteristics	All participants lived in the North Trøndelag region in Norway and were 58 years or older.
Recruitment	Participants were recruited from the North Trøndelag region in Norway. All inhabitants in the defined geographical region were eligible for participation in the HUNT study. For this study, participants aged 58-69.9 years were recruited from HUNT3 and participants aged 70+ years were recruited from HUNT4.
Ethics oversight	This study was approved by the Regional Committee for Medical and Health Research Ethics in Norway (REC Southeast C 565876) as well as according to the General Data Protection Regulation by the Norwegian Agency for Shared Services in Education and Research (SIKT 585403). Participation in HUNT required informed consent, which was provided after receiving oral and written information about the health survey. In participants with reduced capacity to consent, their next of kin gave consent.

Note that full information on the approval of the study protocol must also be provided in the manuscript.

Field-specific reporting

Please select the one below that is the best fit for your research. If you are not sure, read the appropriate sections before making your selection.

☒ Life sciences ☐ Behavioural & social sciences ☐ Ecological, evolutionary & environmental sciences

For a reference copy of the document with all sections, see nature.com/documents/nr-reporting-summary-flat.pdf

Life sciences study design

All studies must disclose on these points even when the disclosure is negative.

Sample size	Biomarker status from all participants in HUNT4 70+ with available plasma were analysed (n=8,949).<br>Plasma biomarker analysis from participants in HUNT3 was restricted to n=2,537 because of funding reasons.
Data exclusions	No data was excluded.
Replication	All laboratory analyses were subject to standard Quality Control (QC) procedures. Information is included in the Methods section regarding QC of the SIMOA assays specifically. Replication of the results was not feasable because of the high number of samples.
Randomization	Not applicable to this type of study as it was a observational study and no interventions were assigned.
Blinding	Laboratory personnel performing plasma pTau217 analysis were blinded to all demographic, clinical and cognitive information on participants.

Behavioural & social sciences study design

All studies must disclose on these points even when the disclosure is negative.

Study description	
Research sample	
Sampling strategy	
Data collection	
Timing	
Data exclusions	

Non-participation

Randomization

Ecological, evolutionary & environmental sciences study design

All studies must disclose on these points even when the disclosure is negative.

Study description

Research sample

Sampling strategy

Data collection

Timing and spatial scale

Data exclusions

Reproducibility

Randomization

Blinding

Did the study involve field work? ☐ Yes ☐ No

Field work, collection and transport

Field conditions

Location

Access & import/export

Disturbance

Reporting for specific materials, systems and methods

We require information from authors about some types of materials, experimental systems and methods used in many studies. Here, indicate whether each material, system or method listed is relevant to your study. If you are not sure if a list item applies to your research, read the appropriate section before selecting a response.

Materials & experimental systems

n/a	Involved in the study	
☐	☒	Antibodies
☒	☐	Eukaryotic cell lines
☒	☐	Palaeontology and archaeology
☒	☐	Animals and other organisms
☐	☒	Clinical data
☒	☐	Dual use research of concern
☒	☐	Plants

Methods

n/a	Involved in the study	
☒	☐	ChIP-seq
☒	☐	Flow cytometry
☒	☐	MRI-based neuroimaging

Antibodies

Antibodies used: ALZpath p-Tau 217 Advantage PLUS, Quanterix

Validation: The study used a two cut-off approach to categorize individuals as Alzheimer's disease neuropathology negative, intermediate, or

Validation	positive. Cut-offs were validated by Ashton et al (doi:10.1001/jamaneurol.2023.5319). The lower cut-off was 0.40 pg/mL (95% sensitivity) and the upper cut-off was ≥ 0.63 pg/mL (95% specificity).

Eukaryotic cell lines

Policy information about cell lines and Sex and Gender in Research

Cell line source(s)	
Authentication	
Mycoplasma contamination	
Commonly misidentified lines (See ICLAC register)	

Palaeontology and Archaeology

Specimen provenance	
Specimen deposition	
Dating methods	

☐ Tick this box to confirm that the raw and calibrated dates are available in the paper or in Supplementary Information.

Ethics oversight	

Note that full information on the approval of the study protocol must also be provided in the manuscript.

Animals and other research organisms

Policy information about studies involving animals; ARRIVE guidelines recommended for reporting animal research, and Sex and Gender in Research

Laboratory animals	
Wild animals	
Reporting on sex	
Field-collected samples	
Ethics oversight	

Note that full information on the approval of the study protocol must also be provided in the manuscript.

Clinical data

Policy information about clinical studies

All manuscripts should comply with the ICMJE guidelines for publication of clinical research and a completed CONSORT checklist must be included with all submissions.

Clinical trial registration	NCT06719453
Study protocol	Information on HUNT can be found on https://hunt-db.medisin.ntnu.no/hunt-db/
Data collection	The Trøndelag Health Study (HUNT) is a population-based health study conducted in the Trøndelag region of Central Norway and has so far spanned four waves (HUNT1-4). Data used in this study was collected as part of HUNT wave 3 (10/2006-6/2008) and wave 4 (9/2017-2/2019). Data collected included demographic information, clinical information and blood samples. Additionaly, in HUNT4, all residents aged 70 years or older were invited to the substudy HUNT4 70+ for a standardized cognitive assessment and diagnosis. Trained health personnel assessed the participants' cognitive, neuropsychiatric, and functional status using standardized clinical scales at a field station, at homes, or in nursing homes. Clinical and research experts made diagnoses on cognition by clinical consensus method.<br>This study collected data on plasma pTau217, with biomarker analysis conducted 1-8/2024, and otherwise used previously collected data.
Outcomes	The outcome was the plasma pTau217 concentration. Plasma pTau217 concentrations were measured using the ALZpath p-Tau 217 Advantage PLUS kit and the Simoa HD-X instrument (both Quanterix - Billerica, MA, USA). We used a two cut-off approach to categorize individuals as Alzheimer's Disease Neuropathological Changes (ADNC) negative, intermediate, or positive, using a lower

cut-off of 0.40 pg/mL (95% sensitivity) and an upper cut-off of ≥ 0.63 pg/mL (95% specificity). We used the upper cut-off to determine with high specificity those individuals with the presence of ADNC (those above the upper cut-off), and the lower cut-off to identify those with a high likelihood of not having ADNC (those below the lower cut-off). The concentrations between the two cut-offs were not able to inform on the presence of ADNC.

Dual use research of concern

Policy information about dual use research of concern

Hazards

Could the accidental, deliberate or reckless misuse of agents or technologies generated in the work, or the application of information presented in the manuscript, pose a threat to:

No	Yes	
☐	☐	Public health
☐	☐	National security
☐	☐	Crops and/or livestock
☐	☐	Ecosystems
☐	☐	Any other significant area

Experiments of concern

Does the work involve any of these experiments of concern:

No	Yes	
☐	☐	Demonstrate how to render a vaccine ineffective
☐	☐	Confer resistance to therapeutically useful antibiotics or antiviral agents
☐	☐	Enhance the virulence of a pathogen or render a nonpathogen virulent
☐	☐	Increase transmissibility of a pathogen
☐	☐	Alter the host range of a pathogen
☐	☐	Enable evasion of diagnostic/detection modalities
☐	☐	Enable the weaponization of a biological agent or toxin
☐	☐	Any other potentially harmful combination of experiments and agents

Plants

Seed stocks

Novel plant genotypes

Authentication

ChIP-seq

Data deposition

☐ Confirm that both raw and final processed data have been deposited in a public database such as GEO.

☐ Confirm that you have deposited or provided access to graph files (e.g. BED files) for the called peaks.

Data access links
May remain private before publication.

Files in database submission

Genome browser session
(e.g. UCSC)

Methodology

Replicates

Sequencing depth

Antibodies

Peak calling parameters

Data quality

Software

Flow Cytometry

Plots

Confirm that:

- [ ] The axis labels state the marker and fluorochrome used (e.g. CD4-FITC).
- [ ] The axis scales are clearly visible. Include numbers along axes only for bottom left plot of group (a 'group' is an analysis of identical markers).
- [ ] All plots are contour plots with outliers or pseudocolor plots.
- [ ] A numerical value for number of cells or percentage (with statistics) is provided.

Methodology

Sample preparation

Instrument

Software

Cell population abundance

Gating strategy

- [ ] Tick this box to confirm that a figure exemplifying the gating strategy is provided in the Supplementary Information.

Magnetic resonance imaging

Experimental design

Design type

Design specifications

Behavioral performance measures

Acquisition

Imaging type(s)

Field strength

Sequence & imaging parameters

Area of acquisition

Diffusion MRI ☐ Used ☐ Not used

Preprocessing

Preprocessing software

Normalization

Normalization template

Noise and artifact removal

Volume censoring

Statistical modeling & inference

Model type and settings

Effect(s) tested

Specify type of analysis: ☐ Whole brain ☐ ROI-based ☐ Both

Statistic type for inference

(See Eklund et al. 2016)

Correction

Models & analysis

n/a	Involved in the study
☐	☐ Functional and/or effective connectivity
☐	☐ Graph analysis
☐	☐ Multivariate modeling or predictive analysis

Functional and/or effective connectivity

Graph analysis

Multivariate modeling and predictive analysis
