# Supplementary material for: Prevalence of Alzheimer’s disease pathology in the community
Source: Nature. 2025 Dec 17;650(8100):182–6. doi: 10.1038/s41586-025-09841-y (PMC12872468; doi:10.1038/s41586-025-09841-y)
Supplement: Supplementary file 3 — Summary of the analytical performance – ALZpath p-Tau 217 HUNT [file 41586_2025_9841_MOESM3_ESM.docx]

Summary of the analytical performance – ALZpath p-Tau 217 HUNT

Calibrators were run in duplicate, and obvious outlier replicates were excluded prior to curve fitting.

Three quality control (QC) levels (human pooled plasma samples) were run in duplicate at the beginning and end of each run.

Repeatability (% CV_r_); Intermediate precision (%CV_Rw_)

At a concentration of 0.4 pg/mL (mean value), the repeatability was 10.3%, and the intermediate precision was 10.3%.

At a concentration of 1.5 pg/mL (mean value), the repeatability was 10.6%, and the intermediate precision was 15.9%.

At a concentration of 1.9 pg/mL (mean value), the repeatability was 8.9%, and the intermediate precision was 15.8%.
